# Maresin-1 suppresses imiquimod-induced skin inflammation by regulating IL-23 receptor expression

**DOI:** 10.1038/s41598-018-23623-9

**Published:** 2018-04-03

**Authors:** Natsuko Saito-Sasaki, Yu Sawada, Emi Mashima, Takashi Yamaguchi, Shun Ohmori, Haruna Yoshioka, Sanehito Haruyama, Etsuko Okada, Motonobu Nakamura

**Affiliations:** Department of Dermatology, University of Occupational and Environmental Health, Kitakyushu, Fukuoka, Japan

## Abstract

The anti-inflammatory effect of omega 3 polyunsaturated fatty acids has been confirmed in various inflammatory disease models. Maresin-1 (MaR1) is a lipid mediator derived from the omega-3 fatty acid docosahexaenoic acid (DHA) that has displayed strong anti-inflammatory effects in various inflammatory disease models. However, the effect of topical MaR1 on cutaneous inflammation remains unclear. Therefore, we initially examined the anti-inflammatory effects of topical Maresin-1 using an imiquimod (IMQ)-induced psoriasis-like mouse model of inflammation. Topical MaR1 reduced the ear swelling response as seen in histological findings. RT-PCR and flow cytometry analyses revealed MaR1 had no inhibitory effect on IL-23, but MaR1 suppressed IL-17A production by γδTCR^mid+^ and CD4^+^ cells in the skin. These inhibitory effects were also observed in a subcutaneous IL-23-injected psoriasis model. MaR1 downmodulated IL-23 receptor (IL-23R) expression by suppressing retinoic acid-related orphan receptor γt (RORγt) expression and internalization in a clathrin-dependent manner in γδTCR^mid+^ and CD4^+^ cells. These results lead to assumptions that topical MaR1 may be a new therapeutic agent for psoriasis and other IL-17-mediated cutaneous inflammatory diseases.

## Introduction

Lipid mediators act on various physiological or pathological conditions by regulating lipid metabolism, cell signaling, and inflammation^1,2^. Previous epidemiological studies showed that omega-3 polyunsaturated fatty acids (PUFAs), which are enriched in fish oils, have strong anti-inflammatory effects^3^. Docosahexaenoic acid (DHA) is a major omega-3 PUFA and its derivatives exert inhibitory effects on various inflammatory diseases^4,5^. Feeding DHA attenuates contact hypersensitivity response by inhibiting inflammatory cell infiltration and interferon-γ (IFN-γ) production in the skin^5^. Furthermore, DHA treatment suppresses atopic skin inflammation through activation of transforming growth factor-β (TGF-β) and interleukin-10 (IL-10), leading to the conversion of CD4^+^ T cells to CD4^+^ FoxP3^+^ regulatory T cells^4^. These findings suggest that DHA has the potential to suppress various cutaneous inflammatory conditions.

Psoriasis is a major cutaneous chronic inflammatory disease, which is clinically characterized by raised, erythematous plaques covered with silvery white scales. Although the detailed pathogenesis mechanisms of psoriasis have been unknown for a long time, the IL-23/IL-17 axis has recently been identified as a major pathogenic cascade in psoriatic inflammation^6^. Since IL-23 from tumor necrosis factor-α (TNF-α) and inducible nitric oxide synthetase (iNOS)-producing dendritic cells (TIP-DCs) is required for maintaining IL-17-producing cells through an autocrine mechanism via TNF-α, the role of these cytokine networks in psoriasis pathogenesis have been shown by the therapeutic effectiveness of cytokine-blocking biologics. Because a beneficial impact of DHA on psoriasis has been identified^7^, it is assumed that DHA or its derivatives might inhibit the IL-23/IL-17 axis in inflammatory diseases.

Among the specialized pro-resolving lipid mediators (SPMs), Maresin 1 (MaR1) has inhibitory effects on experimental inflammatory disease models^8,9^ MaR1 consistently protects mice against experimental colitis by inhibiting the NF-κB pathway, and consequently, inflammatory cytokines^8^. MaR1 also ameliorates lipopolysaccharide (LPS)-induced lung injury in mice by inhibiting neutrophil adhesion and decreasing the production of pro-inflammatory cytokines^9^. In addition, MaR1 exhibits a protective effect against sepsis by reducing LPS and bacteria burden in serum, inhibiting inflammation response, and improving vital organ function^10^. Nevertheless, an inhibitory effect of MaR1 on cutaneous inflammation has not been elucidated.

In this study, we focused on the anti-inflammatory effects of MaR1 on skin inflammation using imiquimod (IMQ) in an IL-23 injection-induced psoriasis model. MaR1 resolves IL-17A production via downmodulating IL-23R expression on γδT cells and CD4^+^ T cells in the skin.

## Results

### MaR1 inhibits IMQ-induced skin inflammation

**Figure 1:**
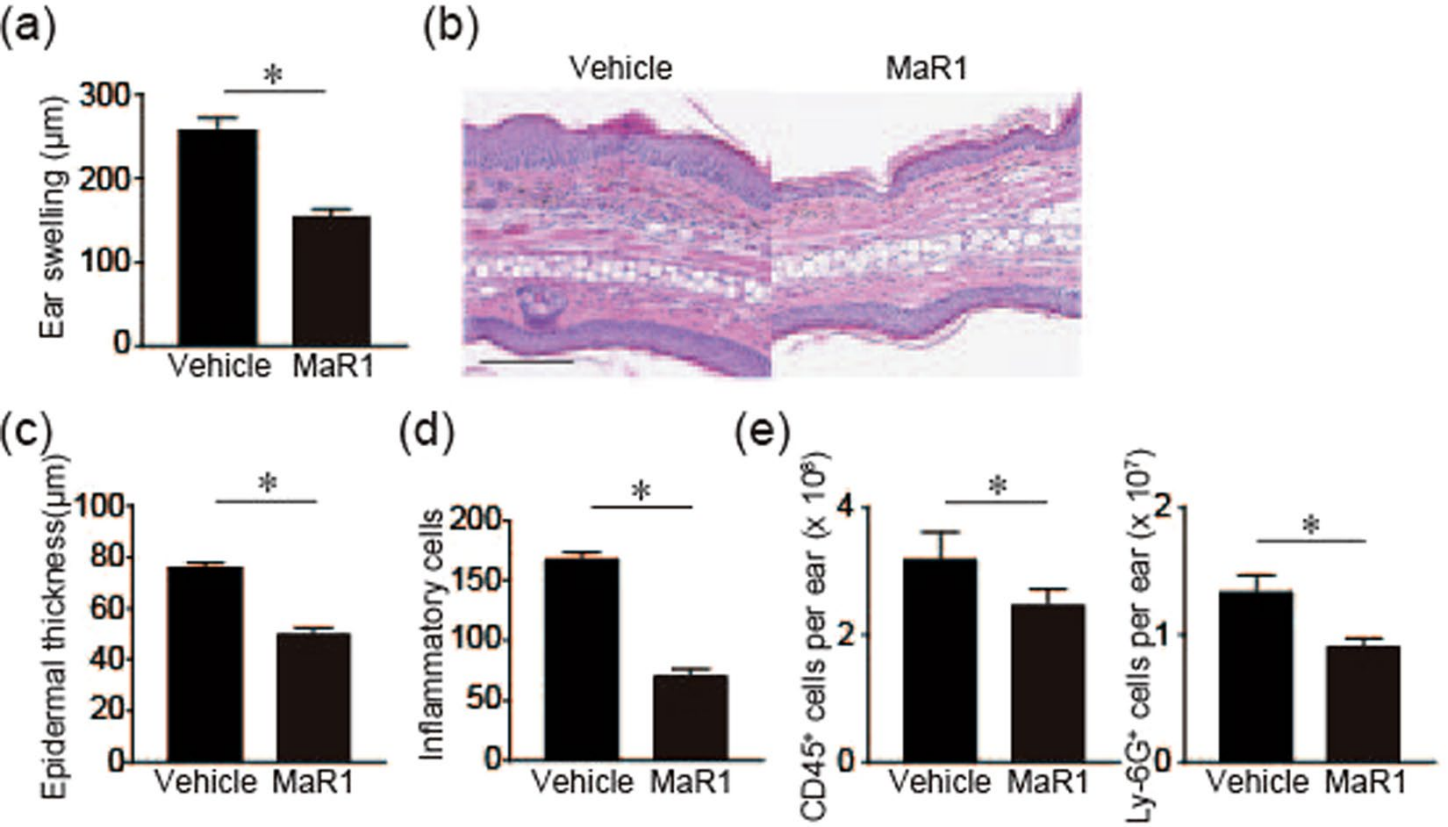
MaR-1 inhibits imiquimod (IMQ)-induced skin inflammation (**a**). Ear swelling. IMQ cream was applied on both ears for 5 consecutive days and ear thickness was measured before and after IMQ application on day 6. The mean ear thickness in both ears was calculated in 6 mice. (**b**) Hematoxylin and eosin staining. Bar, 100 μm. (**c**,**d**) Histological examination. (**c**) Epidermal thickness and (**d**) the number of inflammatory cell infiltration in the skin (n = 6). (**e**) The number of CD45^+^ cells and Ly-6G^+^ cells in the skin. The profiles of inflammatory cells in the skin were subjected to flowcytometry 24 h after IMQ application for 2 days (n = 6). Results are expressed as the mean ± SEM. All *p*-values were obtained by Student’s t test: *P < 0.05. All data are representative of three independent experiments with reproducible results.

IMQ is used for treating actinic keratosis and genital warts through an agonistic effect on Toll-like receptor (TLR) 7 and TLR8. On the other hand, there has been a reported case that IMQ application exacerbated psoriatic skin inflammation in a patient with pre-existing psoriasis during treatment for superficial basal cell carcinoma^11^. Based on this finding, the topical application of IMQ has been used to develop a novel psoriasis mouse model^12^. First, we examined the anti-inflammatory effect of MaR1 in an IMQ-induced psoriasis model. IMQ was applied to ear skin for 5 consecutive days to ensure the effect of MaR1 throughout the course of disease, and ear swelling response was measured as an indicator of skin inflammation. MaR1-treated mice exhibited a significantly decreased ear swelling response (Fig. 1a). Histological analysis of the ears revealed decreased epithelial hyperplasia, dermal edema, and infiltration of lymphocytes in mice treated with MaR1 (Fig. 1b–d). Accordingly, flowcytometry analysis revealed that CD45+ cells and Ly-6G+ cells in the skin were decreased in MaR1 treated mice (Fig. 1e). These results indicated that MaR1 inhibits IMQ-induced psoriasis-like skin inflammation.

### MaR1 decreased IL-17A production in IMQ-induced skin inflammation

**Figure 2:**
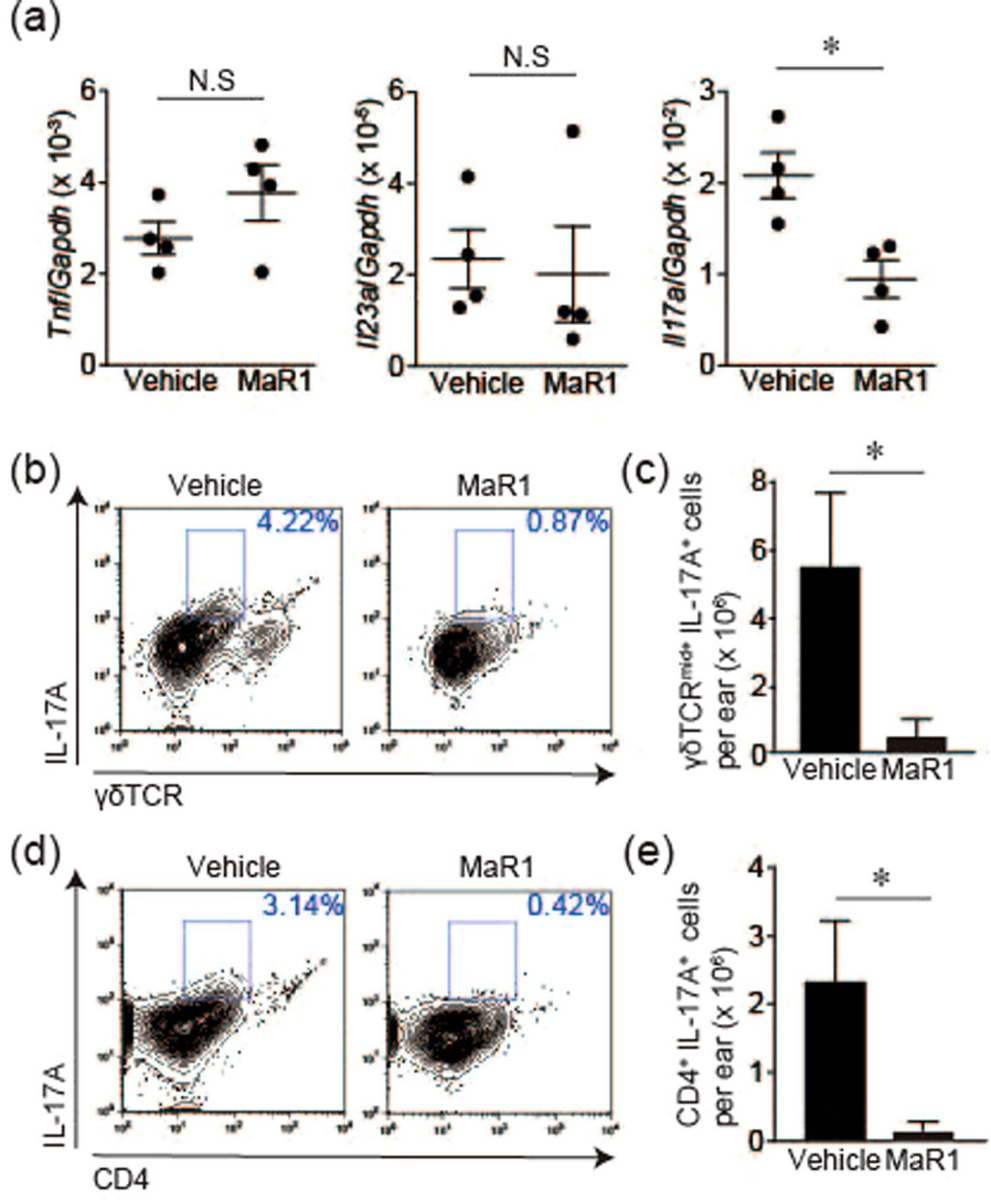
MaR1 impairs the induction of IL-17A in the skin. (**a**) Gene expression in the skin. mRNA was extracted from ear skin 24 h after imiquimod (IMQ) application for two consecutive days, and the gene expressions of *Tnf*, *Il23a* and *Il17a* were analyzed by PCR (n = 4). (**b**) Representative FACS plot of γδTCR^mid+^ IL-17A^+^ cells of ear skin which was corrected 24 h after IMQ application for 2 days. These cells were gated from CD45^+^ cells in skin cell suspensions. (**c**) The number of γδTCR^mid+^ and intracellular IL-17A^+^ cells in the skin (n = 4). (**d**) Representative FACS plot of CD4^+^ IL-17A^+^ cells from ear skin which was collected 24 h after IMQ application for 2 days. These cells were gated from CD45^+^ cells in skin cell suspensions. (**e**) The number of CD4^+^ and intracellular IL-17A^+^ cells in the skin (n = 4). Results are expressed as the mean ± SEM. All *p*-values were obtained by Student’s t test: *P < 0.05. N.S indicates no significant difference. All data are representative of three independent experiments with reproducible results.

IL-23/IL-17 axis is a major signaling cascade in the pathogenesis of psoriasis. IL-23 activates IL-17-producing cells, leading to exacerbation of epidermal hyperplasia and skin inflammation. Since MaR1 had an inhibitory effect on these clinical manifestations confirmed by histological examination (Fig. 1b–d), we speculated that MaR1 might inhibit IMQ-induced skin inflammation by inhibiting the IL-23/IL-17 axis. Accordingly, we next analyzed the effects of MaR1 on inflammatory cytokine compositions in IMQ-induced skin inflammation. The induction of *Il17a* mRNA in MaR1-treated mice was inhibited compared with that in vehicle-treated mice (Fig. 2a). On the other hand, the mRNA expressions of *Il23a* and *Tnf* exhibited no significant difference between vehicle- or MaR1-treated mice. In the skin, the source of IL-17A is mainly classified into two immune cell subsets: γδT cells and CD4^+^ IL-17A-producing cells. Hence, we next examined the inhibitory effect of MaR1 on IL-17A production by these cells. Topical IMQ was applied on both ears for 2 consecutive days, and skin cell suspensions from ear skin were corrected and subjected to flow cytometry analysis on day 3. The major source of IL-17A from cutaneous γδT cells was γδTCR^mid+^ cells. MaR1 inhibited IL-17A production by both γδTCR^mid+^ cells and CD4^+^ cells (Fig. 2b–e). Therefore, MaR1 impaired IMQ-induced skin inflammation through an inhibitory effect on IL-17A production by both γδTCR^mid+^ cells and CD4^+^ cells.

### MaR1 inhibits IL-23-induced skin inflammation via IL-17A blockade

**Figure 3:**
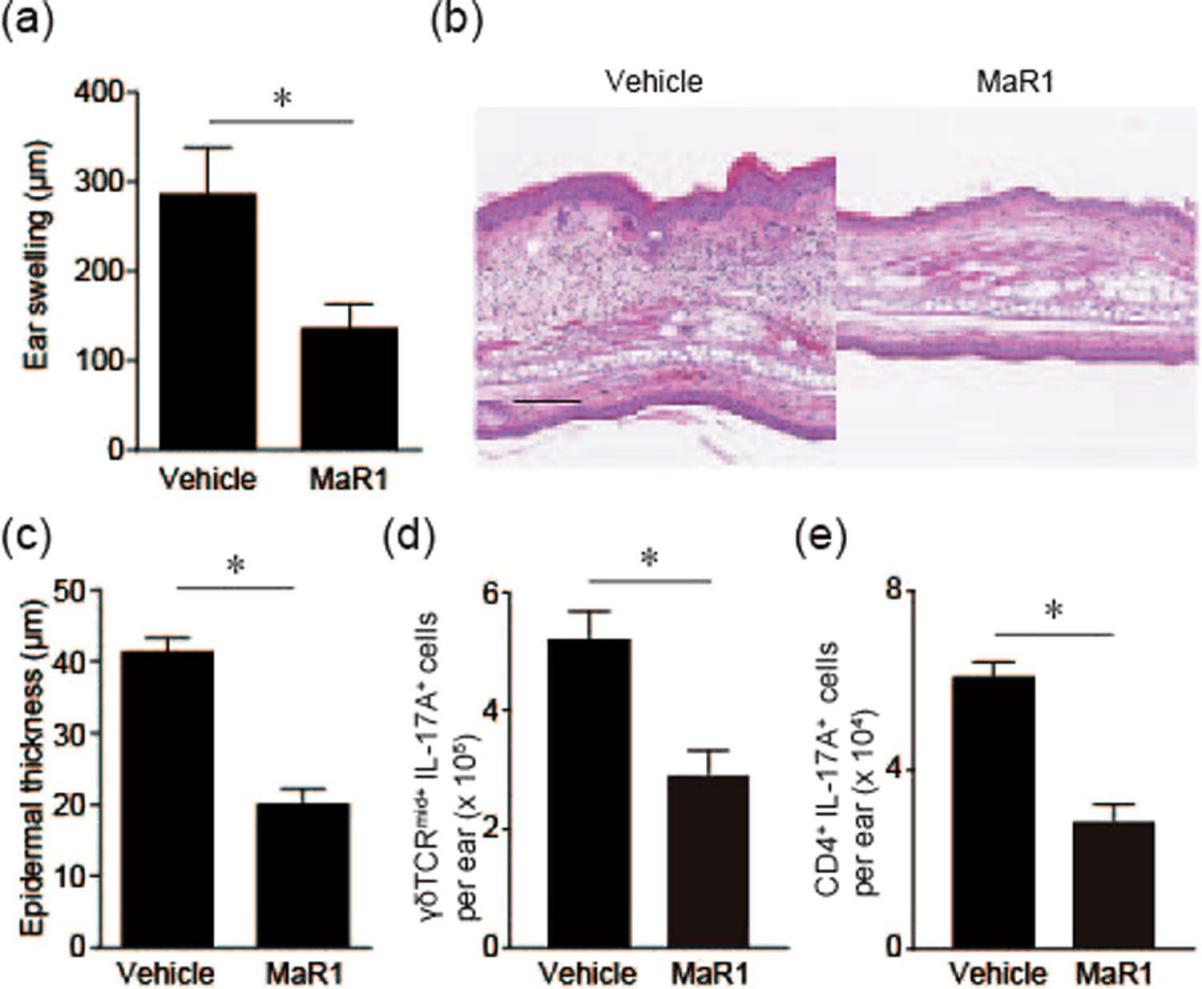
MaR1 inhibits IL-23 injection-induced psoriatic skin inflammation. (**a**) Ear swelling response. Ears swelling was measured from Day 0–16 upon topical application of vehicle or MaR1 (100 ng) 30 min before intradermal injection of 500 ng recombinant mouse IL-23 (n = 3) every other day. The mean ear thickness in both ears was calculated in 3 mice. (**b**) Hematoxylin and eosin-stained sections. Ear skin was collected on day 16. Bar, 200 μm. (**c**) Histological examination of epidermal thickness (n = 3). (**d**) The number of γδTCR^mid+^ and intracellular IL-17A^+^ cells in the skin (n = 4). Ear skin was collected 24 h after IL-23 injection for two consecutive days and subjected to flow cytometry. (**e**) The number of CD4^+^ and intracellular IL-17A^+^ cells in the skin (n = 4). Results are expressed as the mean ± SEM. All *p*-values were obtained by Student’s t test: *P < 0.05. All data are representative of three independent experiments with reproducible results.

It was postulated that IL-23, which is a cytokine driving the development of IL-17-producing Th17 cells, is functionally involved in the pathogenesis of psoriasis. Intradermal injection of IL-23 to mouse skin results in erythema, mixed inflammatory cell infiltration, and epidermal hyperplasia^13^. Because MaR1 inhibits IL-17A production in IMQ-induced skin inflammation, it is necessary to confirm its effect using a more suitable psoriasis model, which is an intradermal IL-23-injected psoriasis model. Consequently, we next analyzed the inhibitory effect of MaR1 on IL-17 production using a psoriasis model of intradermal IL-23 injection. MaR1-treated mice exhibited a significant decrease in the ear swelling response (Fig. 3a). Histological analysis of the ears revealed decreased epithelial hyperplasia in mice treated with MaR1 (Fig. 3b,c). Furthermore, MaR1-treated mice also exhibited decreased IL-17A production by γδTCR^mid+^ cells and CD4^+^ cells in the skin (Fig. 3d,e). Thus, these results indicate that MaR1 also inhibits IL-23-induced skin inflammation by inhibiting IL-17A production by these cells.

### MaR1 inhibits IL-17A production in the skin *in vitro*

**Figure 4:**
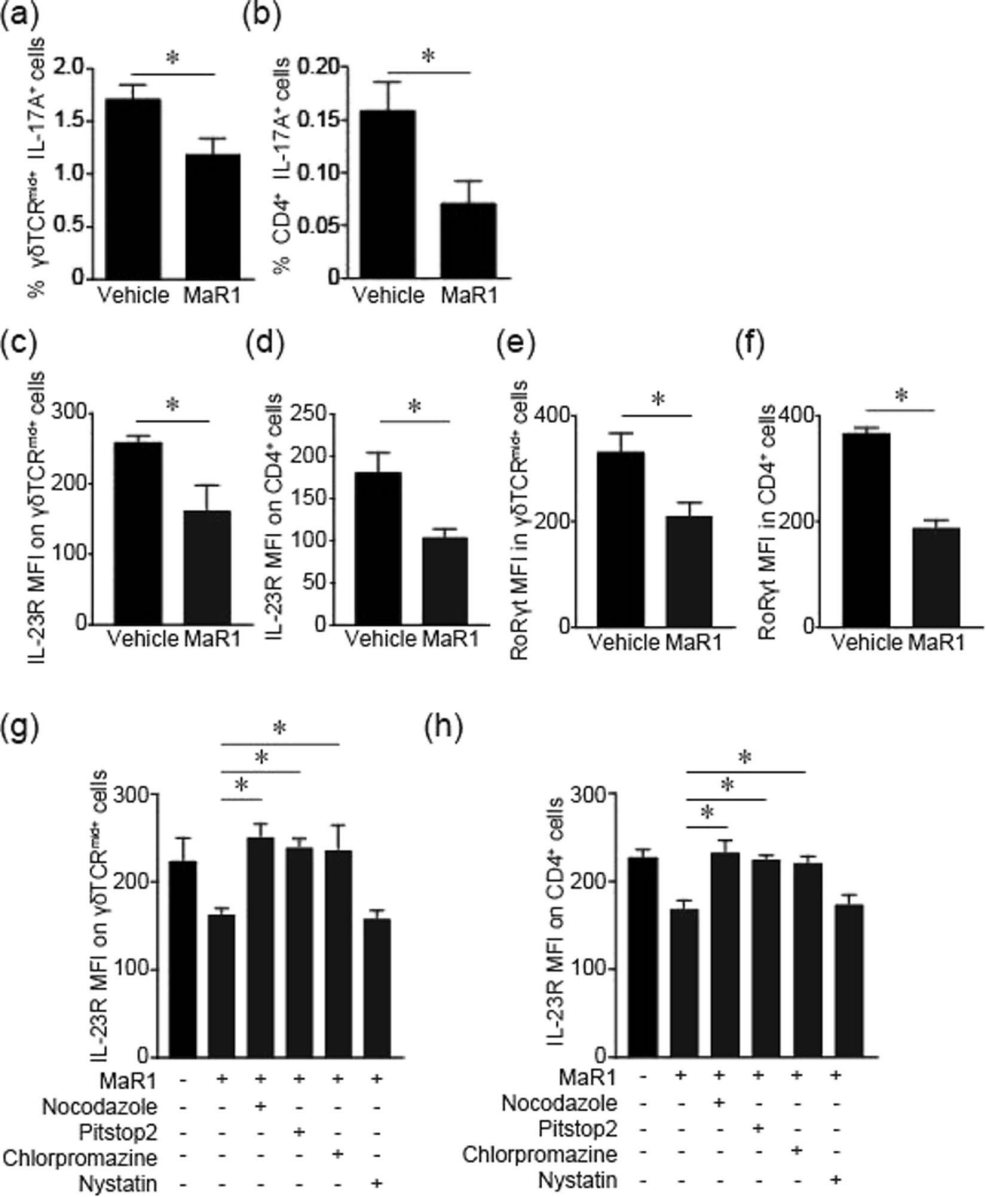
MaR1 treatment downregulates and internalizes IL-23 receptors in an *in vitro* skin assay. The frequencies of γδTCR^mid+^ and intracellular IL-17A^+^ cells in IL-23 stimulated skin cell suspensions (n = 4). Skin cell suspensions were cultured with recombinant IL-23 with vehicle or MaR1 (10 nM) stimulation for 24 h, and the number of γδTCR^mid+^ and intracellular IL-17A^+^ cells were analyzed by flow cytometry. (**b**) The number of CD4^+^ and intracellular IL-17A^+^ cells in in IL-23 stimulated skin cell suspensions (n = 4). (**c**,**d**) The mean fluorescence intensity (MFI) of IL-23R expression on γδTCR^mid+^ (**c**) and CD4^+^ cells (**d**). Skin cell suspensions were cultured with vehicle or MaR1 (10 nM) for 24 h and IL-23R expression was measured by flow cytometry. (**e**,**f**) The MFI of RORγt expression on γδTCR^mid+^ (**e**) and CD4^+^ cells (**f**). Skin cell suspensions were cultured with vehicle or MaR1 (10 nM) for 3 h and intracellular RORγt expression was measured by flow cytometry. (**g**,**h**) The MFI of IL-23R expression on γδTCR^mid+^ (**g**) and CD4 cells (**h**) with internalization inhibitors. Skin suspension cells were cultured with vehicle or MaR1 (10 nM) with nocodazole (10 μM), Pit stop2 (20 μM), nystatin (50 μg/mL), and chlorprozine (25 μg/mL) for 1 h and IL-23R expression was measured by flow cytometry. Results are expressed as the mean ± SEM. All *p*-values were obtained by Student’s t test: *P < 0.05. All data are representative of three independent experiments with reproducible results.

To clarify the detailed inhibitory mechanism of MaR1 on IL-17A production more precisely, we next performed *in vitro* analysis to examine its inhibitory effect on IL-17A production by γδTCR^mid+^ cells and CD4^+^ cells under IL-23 stimulation using cutaneous cell suspensions. As expected, MaR1 suppressed IL-23 stimulated IL-17A production by both γδTCR^mid+^ cells and CD4^+^ cells (Fig. 4a,b). These results suggested that MaR1 attenuates these cell’s response to IL-23 stimulation. We next examined whether MaR1 affects IL-23 receptor (IL-23R) expression on these cells. MaR1 attenuates IL-23R expression on γδTCR^mid+^ cells and CD4^+^ cells compared to vehicle (Fig. 4c,d). Therefore, these results indicate that MaR1 attenuates responses to IL-23 by inhibiting IL-23R expression.

The expression of IL-23R depends on RORγt by which TGFβ and IL-6/IL-21 drive Th17 cell differentiation and this process is orchestrated by TGFβ and IL-6/IL-21^14^. RORγt promotes the expression IL-23R to control the expansion and maintenance of Th17 cells. Therefore, we speculated that MaR1 would regulate RORγt expression and lead to decreased IL-23R expression on these cells. To clarify the possible role of MaR1 on RORγt, skin cell suspensions were treated with vehicle or MaR1 and intracellular RORγt expression was subjected to flow cytometry as mean fluorescence intensity (MFI). RORγt expression was significantly decreased upon MaR1 treatment (Fig. 4e,f). These results indicated that MaR1 inhibits IL-23R expression via inhibiting RORγt.

The additional effect of MaR1 on IL-23R internalization was clarified using internalization inhibitors. Presently, distinct receptor internalization pathways are known: one pathway is via clathrin-coated pits and the other pathway is via caveolae^15^. We examined the effects of nystatin, which disrupts internalization via caveolae^16,17^, and the effects of chlorpromazine and a clathrin-specific inhibitor (Pitstop 2), both of which disrupt internalization via clathrin-coated pits^18,19^. MaR1-induced internalization was significantly inhibited both by chlorpromazine and Pitstop 2, whereas it was completely unaffected by nystatin (Fig. 4g,h). These results indicate that MaR1 promotes clathrin-dependent IL-23R internalization. Therefore, these results indicate that MaR1 inhibits IL-23R expression by regulating RORγt expression and clathrin-dependent IL-23R internalization.

## Discussion

Our study added novel findings that topical MaR1 inhibits IMQ-induced psoriasis in an animal model through inhibition of IL-17A production by both γδTCR^mid+^ and CD4^+^ cells in the skin. Although we could not investigate the detailed regulatory mechanism of MaR1 on RORγt expression, a previous study showed that down-regulating Rorc, which is mediated by the GPR32 and ALX/FPR2 receptors, enhanced generation and function of Foxp3^+^ regulatory T (Treg) cells via the GPR32 receptor^20^. In addition, MaR1 showed a greater inhibitory effect in the IL-23 protocol. The detailed mechanisms remain unclear, the results might indicate a direct inhibition more precisely in the IL-23 protocol than in the IMQ protocol, which exacerbates skin inflammation through various cytokines. Our results also exhibit that MaR1 downregulates IL-23R expression on both γδTCR^mid+^ and CD4^+^ cells, leading to the inhibition of IL-23-mediated skin inflammation. These findings support our results that the DHA derivative MaR1 inhibits IL-17 production in the skin and attenuates psoriatic skin inflammation.

This study indicates that MaR1 suppresses cutaneous IL-17 production. Because another report also showed that MaR1 suppressed the activation of Th17 cells^20^, MaR1 might have a beneficial impact on IL-17-related cutaneous diseases. For example, during the sensitization phase of contact hypersensitivity, IL-17 plays an important role in inducing Th1 in draining lymph nodes. Indeed, since MaR1 also inhibits Th1 differentiation, MaR1 might inhibit contact hypersensitivity. IL-17A has already been revealed as an inducer for Th2 immune responses^21^ in an animal model of atopic dermatitis, and the number of Th17 cells increases in peripheral blood mononuclear cells in patients with atopic dermatitis^22^. Therefore, MaR1 might also attenuate atopic dermatitis skin inflammation by inhibiting IL-17 production in the skin. Furthermore, IL-17A also impairs tight junctions in atopic dermatitis skin^23^. MaR1 maintains the permeability of lung epithelial cells and upregulates the expression of Claudin-1 and ZO-1 after LPS stimulation, leading to decreased lung permeability and reduced lung injury^24^. In atopic dermatitis, these tight junction molecules play an important role against external stimulation. Therefore, MaR1 might also have a beneficial impact on atopic dermatitis by regulating tight junction barriers. Further investigation is necessary to clarify a possible therapeutic candidate of MaR1 for other inflammatory skin diseases.

The detailed mechanism remains unclear, but MaR1 has a clear inhibitory effect on decreased IL-23 response. Dietary omega-3 PUFAs inhibit IL-23R expression in immune cells^25^. Dietary omega-3 PUFAs also decrease obesity-associated Th17 cell-mediated inflammation during colitis by inhibiting RORγt^26^. These findings support our results that MaR1 inhibits IL-17A production by inhibiting IL-23R via downmodulation of RORγt in a clathrin-dependent manner in γδT cells and Th17 cells in the skin.

Our results indicate that MaR1 has the potential to become a novel therapeutic candidate for psoriasis. Thus, our results suggest this study might lead to a new development of treatment for psoriasis.

## Materials and Methods

### Animals and reagents

Female C57BL/6 (B6) mice were purchased from Japan SLC (Hamamatsu, Japan). All experiments were conducted on 8–12-week-old mice. Recombinant mouse IL-23 was purchased from eBioscience (San Diego, CA). MaR1 was purchased from Cayman Chemical (Ann Arbor, MI). Nocodazole, nystatin, and chlorprozine were purchased from Sigma-Aldrich (Poole, UK). Pit stop2 was purchased from Abcam (Cambridge, MA). IMQ was kindly provided by Mochida Pharmaceutical Co., Ltd.

### IMQ-induced psoriasis model

Eight to eleven-week-old mice received a daily topical dose of 62.5 mg of IMQ cream (5%) (Mochida Pharmaceutical, Tokyo, Japan) 30 min after topical application of vehicle (ethanol) or MaR1 (100 ng in 20 μl ethanol/ear) on both ears on each day for 5 consecutive days. The dose of MaR1 was determined by ear swelling response. Thickness of both ears was measured 24 h after the final application by utilizing a thickness gauge (Teclok, Nagano, Japan). The mean ear thickness in both ears was calculated for each mouse.

### Intradermal IL-23 injection

Twenty microliters of PBS, either alone or containing 500 ng recombinant mouse IL-23 from eBioscience (San Diego, CA), was injected intradermally into the ears of anesthetized mice using a 30-gauge needle every other day for 16 days. Topical vehicle (ethanol) or MaR1 (100 ng in 20 μl ethanol/ear) was administrated 30 min before IL-23 injection. Thickness of both mouse ears was measured before injection on day 0 and thereafter on before injections and on day 16. The mean ear thickness of both ears was calculated for each mouse.

### Histological examination

Ears were excised and fixed in 10% formaldehyde, and embedded in paraffin. Sections of 5-μm thickness were prepared and stained with hematoxylin and eosin. Epidermal thickness and the number of infiltrated inflammatory cells were calculated using NanoZoomer (Hamamatsu Photonics, Hamamatsu City, Japan). The number of infiltrated inflammatory cells were calculated from five HPFs (×400) in the dermis of each section.

### Flow cytometry

The ear skin was incubated for 1 h in 10 mL of RPMI (RPMI 1640; Sigma-Aldrich, St Louis, MO), containing 10% heat-inactivated fetal calf serum (FCS, Invitrogen), 50 μM 2-mercaptoethanol, 2 mM l-glutamine, 25 mM N-2-hydroxyethylpiperazine-N′-2-ethanesulfonic acid, 1 mM nonessential amino acids, 1 mM sodium pyruvate, 100 U/mL penicillin, and 100 μg/mL streptomycin) containing collagenase II (Worthington Biochemical., Freehold, NJ) and DNaseI (Sigma-Aldrich). The cell suspensions were filtered with a 40-μm cell strainer and stained with the indicated antibodies.

Cells were stained with various combinations of fluorescence-conjugated mAbs and analyzed with a FACSCanto flow cytometer (BD Biosciences, San Diego, CA) and FlowJo software (TreeStar, San Carlos, CA). The expression levels of cell surface and intracytoplasmic cytokines were analyzed using the following antibodies: FITC-conjugated anti-γδTCR mAb (eBioscience), PE-conjugated anti-CD4 mAb (eBioscience), PE-conjugated anti-Ly-G6 mAb (eBioscience) and anti-IL-23R (Biolegend) mAbs; PerCP-cy5.5-conjugated anti-CD45 mAb (eBioscience); PE-Cy5-conjugated anti-CD4 mAb (eBioscience); and PE-Cy7-conjugated anti-IL-17A mAb (Biolegend). All mAbs were used at a concentration of 1–5 mg per 10^6^ cells, and each incubation was performed for 30 min at 4 °C, followed by two washes in PBS supplemented with 5% FCS and 0.02% sodium azide. For intracellular cytokine staining, cells were incubated in the presence of Goldi Stop (BD Biosciences) for 5 h under stimulation of PMA and ionomycin. Intracytoplasmic cytokines were detected in permeabilized cell suspensions using a BD cytofix/cytoperm Plus Kit (BD Biosciences).

### Quantitative real-time polymerase chain reaction (PCR) analysis

Quantitative real-time PCR analysis was performed as reported previously^27^ with some modifications. Briefly, total RNA was extracted using the Trizol RNA extraction kit (Invitrogen, San Diego, CA). cDNA was reverse transcribed from the total RNA samples using the Prime Script RT reagent kit (Takara Bio, Otsu, Japan). Quantitative real-time PCR was performed by monitoring dsDNA synthesis during the various PCR cycles using SYBR Green I (Takara Bio) and a 7500 Real-Time PCR System (Applied Biosystems, Foster City, CA) according to the manufacturer’s instructions. All primers were obtained from Greiner Japan (Tokyo, Japan). The primer sequences were *Gapdh*, 5′-AGG TCG GTG TGA ACG GAT TTG-3′ (forward), 5′-GGG GTC GTT GAT GGC AAC A-3′ (reverse); *Tnf*, 5′-TGC CTA TGT CTC AGC CTC TTC-3′ (forward) and 5′-GAG GCC ATT TGG GAA CTT CT-3′ (reverse); *Il23a*, 5′-AAC TCC TCC AGC CAG AGG ATC A-3′ (forward), 5′-TCT TGG AAC GGA GAA GGG GG-3′ (reverse); *Il17a*, 5′-CTC CAG AAG GCC CTC AGA CTA C-3′ (forward), 5′-GGG TCT TCA TTG CGG TGG-3′ (reverse). For each sample, triplicate test reactions and a control reaction lacking reverse transcriptase were analyzed to determine gene expression, and results were normalized to levels of the housekeeping *Gapdh* mRNA.

### Statistical analysis

All statistical analyses were carried out using GraphPad Prism 4.0. The Student’s t test was used to calculate statistical differences. All P values less than 0.05 were considered statistically significant.

### Study approval

The murine studies were conducted with the approval of and in accordance with the Guidelines for Animal Experiments of the University of Occupational and Environmental Health.
